# Advanced Messaging Intervention for Medication Adherence and Clinical Outcomes Among Patients With Cancer: Randomized Controlled Trial

**DOI:** 10.2196/44612

**Published:** 2023-08-31

**Authors:** Chen-Xu Ni, Wen-Jie Lu, Min Ni, Fang Huang, Dong-Jie Li, Fu-Ming Shen

**Affiliations:** 1 Department of Pharmacy Shanghai Tenth People’s Hospital Tongji University School of Medicine Shanghai China

**Keywords:** 5G messaging, fifth-generation, medication adherence, patients with cancer, clinical pharmacists, randomized controlled trial

## Abstract

**Background:**

Medication adherence is crucial for improving clinical outcomes in the treatment of patients with cancer. The lack of adherence and adverse drug reactions can reduce the effectiveness of cancer therapy including the quality of life. The commonly used intervention methods for medication adherence continue to evolve, and the age of fifth-generation (5G) messaging has arrived.

**Objective:**

In this study, we conducted a prospective, pilot randomized controlled trial to evaluate the effect of 5G messaging on medication adherence and clinical outcomes among patients with cancer in China.

**Methods:**

The research population was patients with nonsmall cell lung cancer undergoing pemetrexed chemotherapy who require regular folic acid (FA) and vitamin B12 supplements. The intervention and control groups were assigned to 5G messaging and second-generation (2G) messaging, respectively. The patients’ medication adherence and quality of life were assessed at baseline and 1-month and 3-month time points. Moreover, the chemotherapy-related hematologic or nonhematologic toxicities, as well as the serum levels of FA and vitamin B12, were measured.

**Results:**

Of the 567 patients assessed for eligibility between January and May 2021, a total of 154 (27.2%) patients were included. Overall, 80 were randomized to the control group and 74 to the intervention group. The odds of adherence in the 5G messaging intervention group were significantly higher than the control group at the 1-month (62/69, 90% vs 56/74, 76%; adjusted odds ratio 2.67, 95% CI 1.02-7.71) and 3-month (50/60, 83% vs 48/64, 75%; adjusted odds ratio 2.36, 95% CI 1.00-5.23) time points. Correspondingly, the FA and vitamin B12 serum levels of patients in the 5G messaging group were higher than those of the control group. Regarding hematologic toxicities, only the incidence of leukopenia in the intervention group was lower than that in the control group (25/80, 31% in the control group vs 12/74, 16% in the intervention group; *P*=.04). There were no differences in nonhematologic toxicities and quality of life between the 2 groups.

**Conclusions:**

In summary, we conclude that compared with conventional 2G text-based messaging, a 5G messaging intervention can better improve medication adherence and clinical outcome among patients with cancer.

**Trial Registration:**

Chinese Clinical Trial Registry ChiCTR2200058188; https://www.chictr.org.cn/showproj.html?proj=164489

## Introduction

Medication adherence is crucial for improving clinical outcomes in the treatment of patients with cancer. Poor adherence is associated with disease progression and worse survival. Prolonged survival and symptom palliation are the main therapeutic goals. However, the lack of adherence can reduce the effectiveness of therapy including quality of life (QoL) [1] and increase health care costs [2]. Moderate enhancement or at least maintenance of QoL play a vital role among patients with cancer, but QoL may be affected by the severity and frequency of adverse drug reactions. These adverse effects can compromise QoL, increase financial costs, diminish adherence to treatment, and cause medical complications [3,4]. The toxicities of oral cancer therapy include fatigue, nausea, and diarrhea, and the lack of regular contact with an oncology team may impact adherence to oral regimens [5].

In addition to antitumor drugs, supplementation also plays an important role in the treatment and prevention of tumors: for example, the reduction of new skin cancer cases in recipients of lung transplants who take omega-3 fatty acid supplements [6]. In patients with nonsmall cell lung cancer (NSCLC), vitamin D supplementation may improve the survival of patients with early-stage lung adenocarcinoma with lower 25-hydroxy vitamin D levels [7]. However, the intervention management of antitumor drugs and supplementation adherence is challenging. Pemetrexed is the preferred drug for use as a component of platinum-based doublet chemotherapy for patients with NSCLC, because pemetrexed is an antifolate drug that acts primarily by disrupting folate-dependent metabolism and inhibiting multiple enzymes involved in pyrimidine and purine synthesis. Myelosuppression in hematotoxicity is the principal toxicity of pemetrexed. It has been demonstrated that the addition of vitamin B12 and folic acid (FA) to pemetrexed-containing chemotherapy regimens leads to a reduction of severe adverse events, especially hematologic toxicity, without diminishing antitumor efficacy [8]. However, FA supplement is regularly ignored by patients after discharge [9]. Patients who were nonadherent to FA supplement prescriptions had low FA intakes and serum folates, as well as high homocysteine levels and hematologic toxicities [10]. Thus, it is necessary to manage the medication adherence of patients with NSCLC undergoing pemetrexed chemotherapy who require FA and vitamin B12 supplements.

Currently, the commonly used intervention methods for medication adherence include a variety of medical-related text messaging interventions, apps, websites, etc. All of them have achieved good results in the intervention of tumor medication adherence [11-13]. Nevertheless, another study has found that text messaging failed to improve any outcomes in patients with breast cancer [14]. Smartphone apps require complex operations, such as downloading the app, and both apps and web-based education platforms possess spatial and temporal limitations—they depend on Wi-Fi or data networks to send high-definition videos to patients. With the development of information technology, second-generation (2G) text-based messaging has been raised to fifth-generation (5G) messaging. 5G messaging is constructed based on the latest standards of the Global System for Mobile Communications Association to achieve multimedia and interactive messages, which have the advantages of high speed, low delay, and greater connectivity.

5G messaging is superior in many ways to the commonly used intervention methods for medication adherence. Compared with the conventional 2G text-based messaging, advanced 5G messaging support multiple media formats, including high-definition pictures, audio, video, and emoticons; geographic location; contact card, etc. The video and audio can be delivered in many ways (including via email or websites). However, most patients with chronic conditions, including patients with cancer, are older adults, and using a mobile phone with 5G network connection is more convenient for the management of medication adherence among older adults.

More than 97% of county towns and 40% of urban areas in China have been covered by 5G networks. 5G applications are accelerating in areas such as education, health care, and information consumption. More than 600 tertiary hospitals in China have launched 5G+ emergency, remote diagnosis, and health management applications [15]. The application of 5G in distance education has gained attention, enabling patients in rural areas to obtain the same medical and pharmaceutical services as those in urban areas. By connecting a smartphone to the 5G messaging service of a communication company, the operators can supply personalized services and consultations to users through abundant media methods. Users can easily enjoy the closed-loop administration through click interaction and multimedia without complex operations and Wi-Fi–dependent limitations.

Therefore, we conducted this pilot study to assess whether a pharmacist-lead 5G messaging intervention can enhance medication adherence of regular FA supplementation and thus improve clinical outcomes among patients with NSCLC. This is the first study to apply 5G messaging to medication adherence among patients with cancer.

## Methods

### Research Setting

We conducted a randomized controlled trial with the concealment of allocation and single-blinded outcome assessment. The study was performed from January to May 2021 at Shanghai Tenth People’s Hospital, which is the cancer center affiliated to Shanghai Tongji University. The study was registered in the Chinese Clinical Trial Registry (ChiCTR2200058188).

### Participants Enrollment

We included patients who (1) were diagnosed with cytologically or histopathologically proven NSCLC and planned for upfront pemetrexed-platinum doublet chemotherapy; (2) owned a smartphone and were able to communicate in Mandarin Chinese; (3) had the capability to read messages and watch videos; and (4) had an Eastern Cooperative Oncology Group (ECOG) score of 0-3.

We excluded patients who (1) were diagnosed with other cancers; (2) did not speak Mandarin Chinese or were using a mobile phone that was unable to receive 5G messaging; (3) had reading or comprehensive impairments; and (4) were unwilling to participate in the trial.

### FA and B12 Supplementation

Recommendations for supplementation included starting oral FA (350-1000 μg daily) 1 week before the first dose of pemetrexed and continuing the same for at least 2 weeks beyond the end of pemetrexed treatment. Along with FA, intramuscular vitamin B12 injection (1000 μg) should be administered and repeated every 9 weeks until the cessation of treatment [16].

### 5G Messaging Intervention

Randomization was performed in advance using a web-based random number generator [17] in a 1:1 ratio. The control group received 2G messaging (text only) twice a week. The text is as follows: “Dear <Patient Name>, please be reminded to take folic acid tablets as instructed by your doctor/pharmacist. Take tablets (350~1000 μg, usually 400 μg) daily starting one week before the dose of pemetrexed and continuing the same for at least 21 days beyond end of pemetrexed.” This message was in Chinese.

The intervention group received 5G messaging twice a week. The contents of the 5G messaging intervention consisted of not only text but also video and audio messages of medication education: (1) the text content is the same as that from 2G messaging, with “For detailed explanation, please watching the following video or audio” added to the end; (2) the video content (Multimedia Appendix 1) was made according to the prescribing information of pemetrexed [16] and the guideline for FA supplementation in China [18]; (3) the video content has also been synchronized with the production of an audio version with only sound but no video (Multimedia Appendix 2). The video and audio messages were in Chinese (see Multimedia Appendix 3 for an English translation of the video content). The participants had the option to stop the messaging intervention at any time. The pharmacist followed up all patients by conducting surveys via phone calls every month.

### Measurement and Outcomes

We collected data using the following instruments: (1) the Morisky Medication Adherence Scale 8 item (MMAS-8) [19-21]; (2) the EuroQol EQ-5D-3L; (3) the Beliefs about Medicines Questionnaire (BMQ)–Specific; and (4) a predefined data collection form. The MMAS-8 is an 8-item questionnaire designed to facilitate the identification of barriers and behaviors associated with adherence to medication. The possible answers to questions 1 to 7 are “Yes” (0 points) or “No” (1 point). Five of the questions are scored in reverse. The possible answers to question 8 are “Never,” “Occasionally,” “Sometimes,” “Often,” and “All the time,” scoring 1, 0.75, 0.50, 0.25, and 0 points, respectively [19]. The EuroQol EQ-5D-3L comprises the following 5 dimensions: mobility, self-care, usual activities, pain/discomfort, and anxiety/depression. Each dimension has 3 levels: no problem, some problems, and extreme problems [22]. The BMQ-Specific assesses patients’ beliefs about the particular medications prescribed for them, comprising 2 subscales: Specific Necessity and Specific Concerns. Each item of the BMQ subscales is scored on a 5-point Likert scale ranging from 1 (strongly disagree) to 5 (strongly agree) [23]. In the data collection form, information regarding age, gender, other comorbidities, and concurrently used drugs was collected.

The primary outcomes were (1) the proportion of patients who adhered to medications at the 1-month time point and (2) incidence of any grade of hematologic toxicities (anemia, leukopenia, neutropenia, or thrombocytopenia) and nonhematologic toxicities (neuropathy, fatigue, fever, constipation, diarrhea, and vomiting) according to the National Cancer Institute Common Terminology Criteria for Adverse Events, version 3.0, during the study period. The secondary outcomes were (1) the proportion of patients who adhered to medications at the 3-month time point; (2) changes in serum levels of FA and vitamin B12; and (3) change in QoL from baseline to the 3-month time point.

### Statistical Analysis

To detect a 20% difference in adherence between the control and intervention groups and to account for 20% loss to follow-up, we enrolled at least 152 patients (76 per group). Data were presented as absolute numbers, percentages, means with SDs, or medians with IQRs as appropriate. Characteristics and the QoL of patients randomized to the intervention and control groups were compared using chi-square test for all categorical variables and 2-tailed independent *t* test or Mann-Whitney *U* test for all continuous variables. Univariable logistic regression models were used to estimate the odds ratio (ORs) with 95% CIs of the intervention for adherence outcomes. The change in QoL was assessed using the McNemar test (categorical) and Wilcoxon signed rank test (continuous). All tests considered 2-sided *P* values of ≤.05 to be statistically significant. SPSS Statistics (version 25.0; IBM Corp) and GraphPad Prism (version 7.0; GraphPad Software, Inc) were used for statistical analyses. GraphPad Prism was also used to create graphs.

### Ethics Approval

All study materials and procedures were approved by the institutional review board of Shanghai Tenth People’s Hospital (protocol ID SHSY-IEC-4.1/21-248/01).

### Informed Consent and Compensation

All enrolled participants have signed the informed consent forms before the trial started. They consented to primary data collection and allowed secondary analysis without additional consent. All the study data are anonymous or deidentified. The compensation type for all enrolled participants in human subjects research is cash.

## Results

### Baseline Characteristics

**Figure 1:**
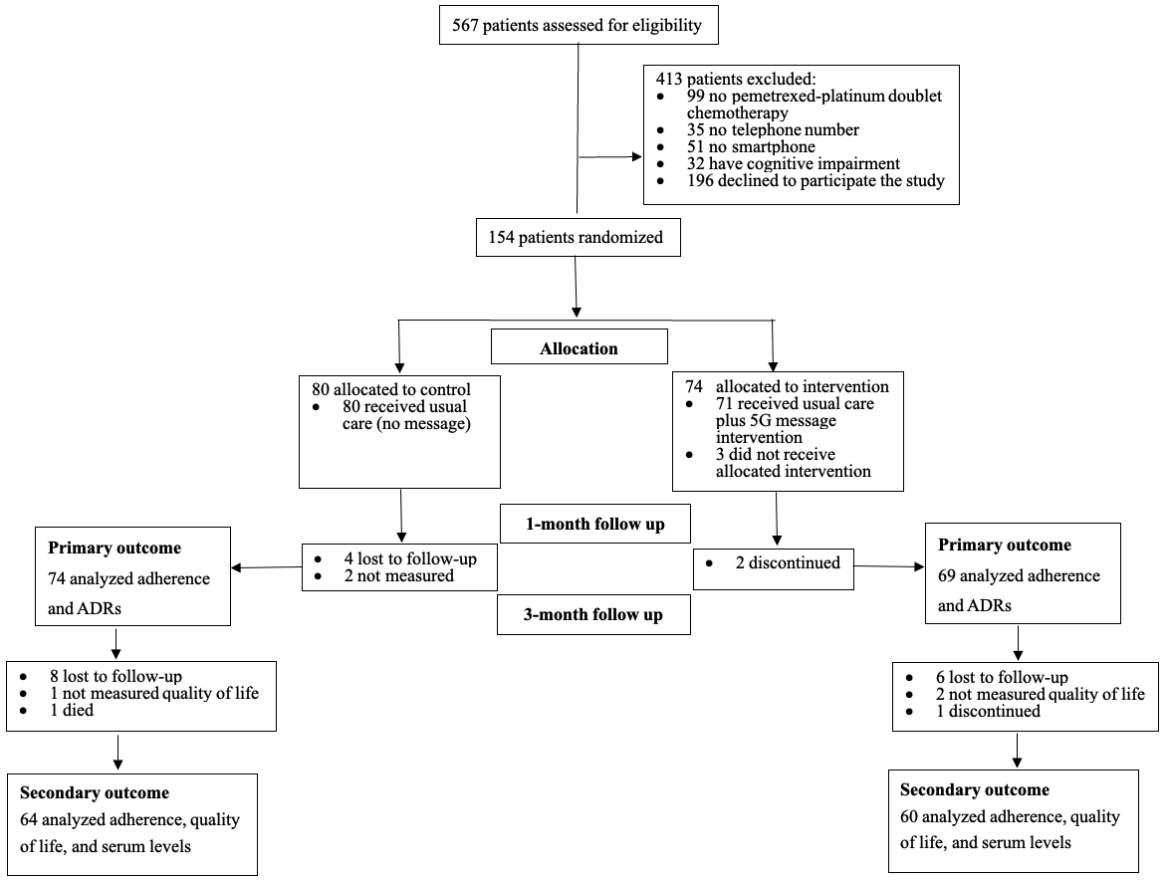
Flowchart of the study population. 5G: fifth generation; ADR: adverse drug reactions.

Of the 567 patients assessed for eligibility between January and May 2021, a total of 154 (27.2%) patients were included; 217 (38.3%) patients were excluded due to exclusion criteria, and 196 (34.6%) declined to participate. Of the 154 patients included, 80 were randomized to the control group and 74 to the intervention group. Three patients in the intervention group did not receive the intervention due to poor health, resulting in 71 patients in the intervention group. Out of the 154 patients, 143 (92.9%) and 124 (80.5%) completed the 1-month and 3-month follow-ups, respectively. The reasons for the loss of follow-up included being out of contact, death, discontinuing the intervention, etc (Figure 1).

The mean age of the patients was 64.3 (SD 8.1) years, and 43.5% (67/154) were male. The proportion of older patients (aged >65 years) was 39.6% (61/154). In addition to cancer, the majority of patients also had comorbidities, mainly hypertension (55/154, 35.7%), diabetes (25/154, 22.7%), and other comorbidities (35/154, 22.7%). Furthermore, 16.9% (26/154) of patients took more than 2 non-antitumor drugs. BMQ Specific Necessity and Concern scores were measured among the patients. No differences in the demographics and general characteristics, medical history, comorbidities, and BMQ-Specific scores were found among the control and intervention groups (Table 1).

**Table 1:** Baseline characteristics of the study population.

Patient characteristic		All patients (n=154)	Control (n=80)	Intervention (n=74)	*P* value
**Demographics and general characteristics**					
	Age (years), mean (SD)	64.3 (8.1)	62.4 (8.4)	66.5 (7.3)	.06^a^
	Aged >65 years, n (%)	61 (39.6)	27 (33.8)	34 (45.9)	.12^b^
	Male, n (%)	67 (43.5)	29 (36.3)	38 (51.4)	.06^b^
**Medical history and comorbidities, n (%)**					
	Hypertension	55 (35.7)	23 (28.8)	32 (43.2)	.06^b^
	Diabetes	35 (22.7)	19 (23.8)	16 (21.6)	.75^b^
	Other comorbidities	35 (22.7)	15 (18.8)	20 (27)	.22^b^
	>2 Non-antitumor drugs	26 (16.9)	12 (15)	14 (18.9)	.52^b^
**BMQ^c^ Specific score, median (IQR)**					
	BMQ Specific Necessity	23 (21-25)	20 (14-24)	21 (19-23)	.94^d^
	BMQ Specific Concern	11 (9-15)	12 (9-15)	10 (9-13)	.29^d^

^a^Independent (2-tailed) *t* test.

^a^Chi-square test.

^c^BMQ: Beliefs about Medicines Questionnaire.

^d^Mann-Whitney *U* test.

### Changes in Measures

**Figure 2:**
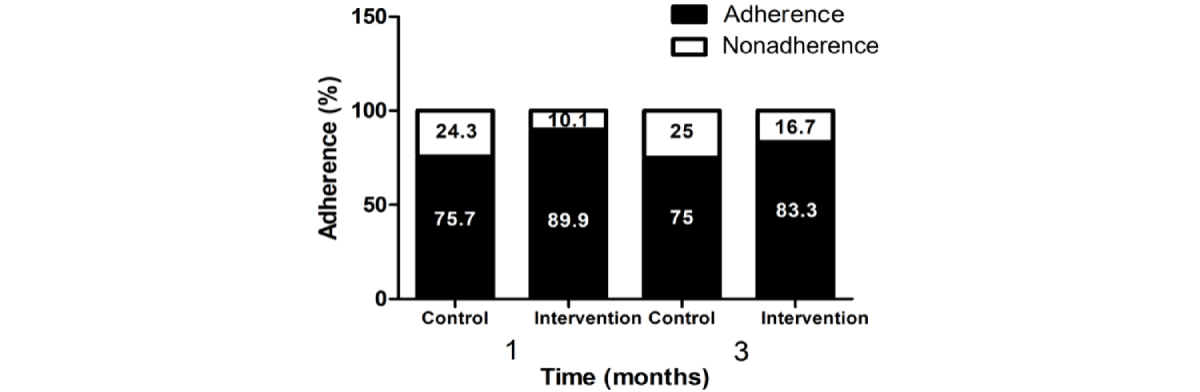
Patient’s medication adherence at the 1-month and 3-month time points. The MMAS-8 Scale, content, name, and trademarks are protected by US copyright and trademark laws. Permission for use of the scale and its coding is required. A license agreement is available from MMAR, LLC., www.moriskyscale.com. MMAS-8: Morisky Medication Adherence Scale 8 item.

The full score of the MMAS-8 is 8 points. A score of <6 represents poor adherence, a score of 6-8 represents moderate adherence, and a score of 8 represents good adherence. A greater proportion of patients were adherent in the intervention group than the control group at the 1-month (62/69, 90% vs 56/74, 76%; adjusted OR 2.67, 95% CI 1.02-7.71) and 3-month (50/60, 83% vs 48/64, 75%; adjusted OR 2.36, 95% CI 1.00-5.23) time points (Figure 2; Table 2).

There were significant increases in median EQ-5D-3L index value from baseline to the 3-month time point in both the control (0.68, IQR 0.54-1 vs 1, IQR 0.726-1; *P*<.001) and intervention (0.76, IQR 0.65-1 vs 1, IQR 0.73-1; *P*=.004) groups. The results indicated that both 2G and 5G messaging have the potential to ameliorate physical and mental health in the QoL. However, there was no significant difference in median change of EQ-5D-3L index values between the control and intervention groups at baseline or the 3-month time point (0.214, IQR 0.000-0.375 vs 0.000, IQR 0.000-0.279; *P*=.08; Table 3).

**Table 2:** Patient adherence at the 1-month and 3-month time points, measured using the MMAS-8^a,b^. Samples exclude patients who were not measured for adherence at the 1-month and 3-month time points.

Time point			Control, n (%)		Intervention, n (%)		Absolute difference in proportions (%; 95% CI)			Univariable analysis			
									OR^c^ (95% CI)			*P* value	
**1 Month (control: n=74 and intervention: n=69)**								14.3 (7-26.5)			2.67 (1.02-7.71)		.03^d^
	Adherence	56 (76)		62 (90)									
	Nonadherence	18 (24)		7 (10)									

**3 Months (control: n=64 and intervention: n=60)**								16 (1.5-28.4)			2.36 (1.00-5.23)		.049^d^
	Adherence	48 (75)		50 (83)									
	Nonadherence	16 (25)		10 (17)									

^a^MMAS-8: Morisky Medication Adherence Scale 8 item.

^b^The MMAS-8 Scale, content, name, and trademarks are protected by US copyright and trademark laws. Permission for use of the scale and its coding is required. A license agreement is available from MMAR, LLC., www.moriskyscale.com.

^c^OR: odds ratio.

^d^*P*<.05.

**Table 3:** Changes in quality of life from baseline to the 3-month time point.

EQ-5D-3L		Baseline^a^		3-month time point^a^		Comparison					
		Control^b^ (n=80)	Intervention^b^ (n=74)	Control^b^ (n=64)	Intervention^b^ (n=60)	*P*_1_ value^c^		*P*_2_ value^d^		*P*_3_ value^e^	
**Mobility, n (%)**							.34^f^		.58^f^		—^g^
	No problems	50 (62)	57 (77)	43 (67)	50 (83)						
	Problems	30 (38)	17 (23)	21 (33)	10 (17)						
**Self-care, n (%)**							.27^f^		.30^f^		—
	No problems	66 (82)	65 (88)	58 (91)	57 (95)						
	Problems	14 (18)	9 (12)	6 (9)	3 (5)						
**Usual activities, n (%)**							.05^f^		.18^f^		—
	No problems	61 (76)	62 (84)	72 (90)	56 (93)						
	Problems	19 (24)	12 (16)	8 (10)	4 (7)						
**Pain/discomfort, n (%)**							.81^f^		.83^f^		—
	No problems	48 (60)	51 (69)	42 (66)	43 (72)						
	Problems	32 (40)	23 (31)	22 (34)	17 (28)						
**Anxiety/depression, n (%)**							.052^f^		.12^f^		—
	No problems	46 (58)	45 (61)	45 (70)	42 (70)						
	Problems	34 (42)	29 (19)	19 (30)	18 (30)						
EQ-5D-3L index value, median (IQR)		0.68 (0.54-1)	0.76 (0.65-1)	1 (0.726-1)	1 (0.73-1)	<.001^h^		.004^h^		—	
Change of EQ-5D-3L index value, median (IQR)		—	—	0.214 (0.000-0.375)	0.000 (0.000-0.279)	—		—		.08^f^	

^a^No differences in each dimension and index value of the EQ-5D-3L between the control and intervention groups at baseline or the 3-month time point.

^b^Sample size of patients who reported EQ-5D-3L at both baseline and the 3-month time points.

^c^*P*_1_: Comparison between baseline and 3-month EQ-5D-3L scores of the control group.

^d^*P*_2_: Comparison between baseline and 3-month EQ-5D-3L scores of the intervention group.

^e^*P*_3_: Comparison between changes from baseline of EQ-5D-3L index values of the control and intervention groups at the 3-month time point.

^f^McNemar test.

^g^Not applicable.

^h^Wilcoxon signed rank test.

In the aspect of hematologic toxicities, 66.5% (102/154) of patients developed anemia (any grade), 35.9% (55/154) developed neutropenia (any grade), 47.6% (73/154) developed leukopenia (any grade), and 30.5% (47/154) developed thrombocytopenia (any grade). There was not statistically less incidence in the intervention group compared with the control group except for leukopenia (25/80, 31% in the control group vs 12/74, 16% in the intervention group; *P*=.04; Table 4). There were not any significant differences in the incidence of nonhematologic toxicities (neuropathy, fatigue, fever, constipation, diarrhea, and vomiting) between the control and intervention groups (all *P*>.05; Table 4).

**Figure 3:**
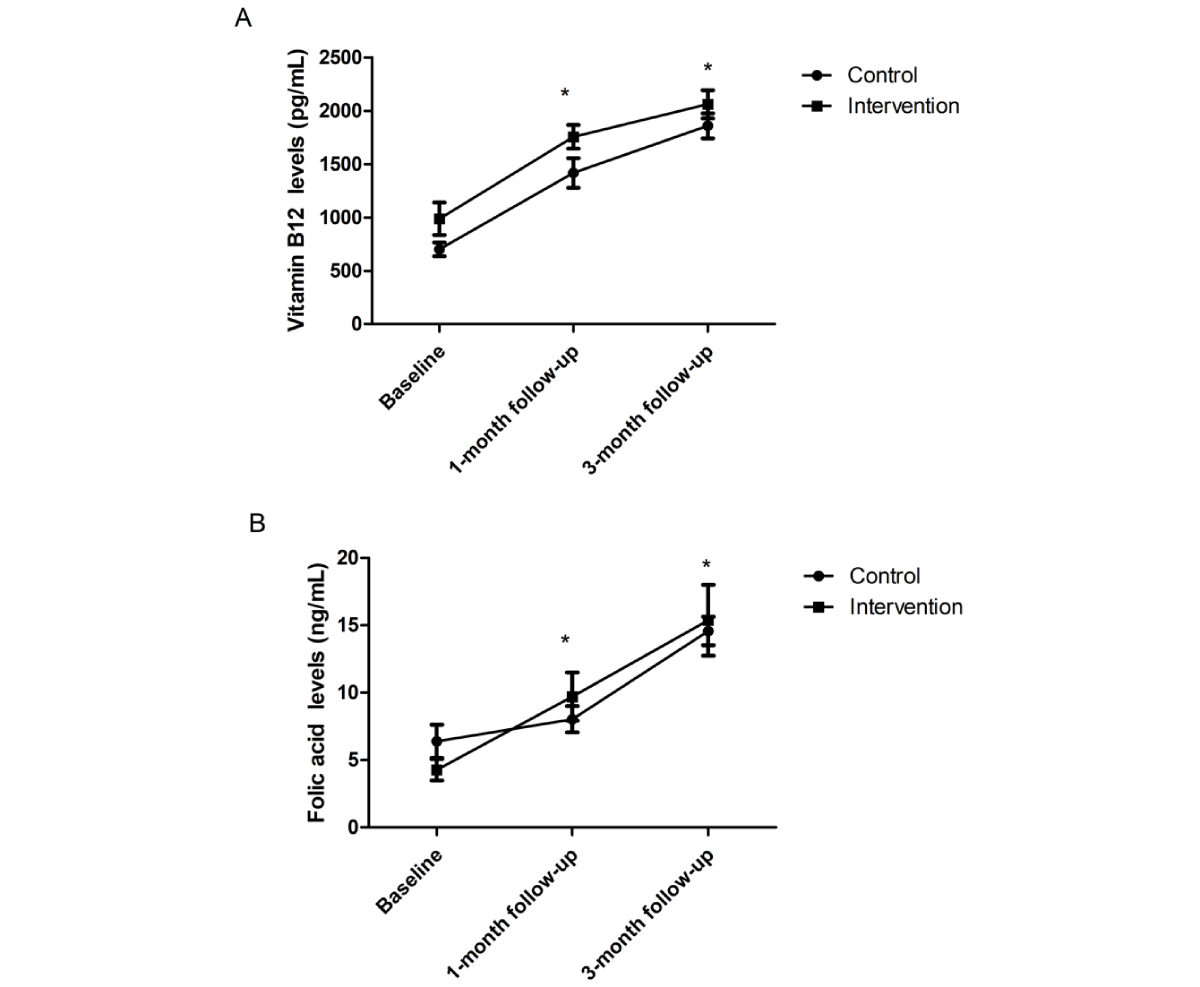
Line and scatter plots illustrate serum levels of vitamin B12 (A) and folic acid (B) in the control and intervention treatment. Circles represent mean values, and error bars represent 95% CIs. *P<.001.

In total, 143 patients (74 control and 69 intervention) contributed to the analysis of the FA and vitamin B12 assays at the 1-month time point, and 124 patients (64 control and 60 intervention) contributed at the 3-month time point. FA and vitamin B12 levels at the 1-month and 3-month time points were significantly higher than the levels at baseline (all *P*<.001). FA and vitamin B12 levels were statistically greater in the intervention group than the control group at the 1-month and 3-month time points (all *P*<.001; Figure 3), which corresponds to the improvement of medication adherence.

**Table 4:** Hematologic and nonhematologic toxicity profiles of the patients.

Profile				Control (n=80), incidence (%; 95% CI)		Intervention (n=74), incidence (%; 95% CI)		*P* value
**Hematologic** **toxicit** **ies**								
	**Anemia**							
		Any grade	37.8 (26.4-49.9)		28.7 (18.6-40.8)		.25	
		Grade 3/4	7.7 (7.4-9.3)		7.0 (6.4-9.2)		.90	
	Leukopenia, any grade			31.4 (21.7-42.1)		16.2 (8.0-27.8)		.04^a^
	Neutropenia, any grade			20.1 (12.5-31.4)		15.8 (7.4-25.8)		.36
	Thrombocytopenia, any grade			18.3 (10.6-28.3)		12.2 (5.1-22.8)		.32
**Nonh** **ematologic** **toxicit** **ies**								
	Neuropathy, any grade			19 (10-31)		21 (12-32)		.84
	Fatigue, any grade			40 (22-58)		38 (20-59)		.89
	Fever, any grade			10 (3-19)		8 (5-17)		.78
	Constipation, any grade			12 (6-23)		7 (3-17)		.43
	**Diarrhea**							
		Any grade	13 (6-22)		12 (6-23)		.99	
		Grade 3/4	5 (2-13)		6 (2-15)		.99	
	**Vomiting**							
		Any grade	17 (9-27)		12 (6-22)		.36	
		Grade 3/4	5 (1-13)		4 (1-12)		.99	

^a^*P*<.05.

## Discussion

### Principal Findings

This pilot study was an attempt by a clinical pharmacist to use 5G technology to innovate and carry out intelligent pharmaceutical care in patients with cancer. Compared with 2G messaging, the 5G messaging intervention enhanced the proportion of adherent patients by over 14.2% at the 1-month time point and 8.3% at the 3-month time point. The levels of FA and vitamin B12 in patients with NSCLC in the 5G messaging group were higher than those in 2G messaging group, which corresponds to the improvement of medication adherence. In the aspect of hematologic toxicities, the incidence of leukopenia in the intervention group was lower than that in the control group. Compared with 2G messaging, the 5G messaging intervention enhanced medication adherence of FA and vitamin B12, which resulted in the partially reduced risk of myelosuppression in patients with NSCLC undergoing pemetrexed chemotherapy.

There were significant increases in median EQ-5D-3L index values from baseline to the 3-month time point in both the control and intervention groups. The results show that both 2G and 5G messaging have the potential to ameliorate physical and mental health in the QoL. Digital video interventions represent effective tools for enhancing mental health [24] and physical activity [25] in patients. The intervention of 5G messaging for physical and mental health needs further research. Recently, researchers have begun to present contents of medical education in videos. Information provided via video may better engage participants and improve their retention of content [26]. There is still debate about whether text or video is more effective as an intervention in health care. Vandelanotte et al [27] found that the personally tailored videos were not more effective than personally tailored text messages in increasing moderate-to-vigorous physical activity.

The increasing availability and ease of use of smartphone apps has allowed for substantial growth of apps that can be used for health behavior change. The mobile app can send text messages, check notifications, and open video channels. The telehealth program is feasible and enhance participants’ and their families’ access and motivation to engage in self-management [28]. However, the use of apps requires it to be downloaded, which would occupy a lot of the memory space of the smartphone. 5G technology, with low latency, high speed, enhanced high resolution, superior reliability, and less energy consumption, is bound to transform telemedicine and the health care industry as a whole [29]. This next-generation wireless networking of 5G technology has many far-reaching implications in both preventive and therapeutic care of the patients.

Huang et al [30] described that patients preferred to have reminders sent 30 minutes before their scheduled time for medication. A review of interventional trials to improve medication adherence stressed that personalized and interactive reminders are the most effective [31]. Our findings showed that 5G messaging played a good guiding role in the medication adherence of patients with cancer. 5G messaging possess interesting and unlimited potential. In the future, the frequency and timing of sending 5G messages shall be set by patients before sending. It is necessary to strengthen the timeliness and personalization of 5G messaging interventions to improve the long-term impact on patients’ medication adherence. 5G technology will hopefully promote the innovation of intelligent pharmaceutical care and improve the efficiency and quality of clinical pharmaceutical care.

### Limitations

Several issues in our study should be considered. First, this was a single-center study with a small sample size, and further studies with larger sample sizes in multiple centers, especially in rural areas, are needed to confirm these results. Second, this pilot study describes the effect of 5G messaging on adherence with supplementation medication for lung cancer. Further large-scale studies are needed to research the effect of 5G messaging on medication adherence to antitumor drugs. Third, the study focused on the Chinese population. As potential cultural differences could alter external validity for the use of 5G messaging, studies in other populations are planned.

### Conclusions

Our randomized controlled trial showed a significant effect of 5G messaging in improving medication adherence among patients with cancer. Future studies could investigate the use of a tailored 5G messaging intervention on clinical outcomes according to the patients’ preference.

## Appendix

Multimedia Appendix 1 The video of medication education in Chinese.

Multimedia Appendix 2 The audio of medication education in Chinese.

Multimedia Appendix 3 English translation of the video content presented in Multimedia Appendix 1.

Multimedia Appendix 4 CONSORT-eHEALTH checklist (V 1.6.1).

## Abbreviations

2G: second-generation
5G: fifth-generation
BMQ: Beliefs about Medicines Questionnaire
ECOG: Eastern Cooperative Oncology Group
FA: folic acid
MMAS-8: Morisky Medication Adherence Scale 8 item
NSCLC: nonsmall cell lung cancer
QoL: quality of life

## Data Availability

The data sets generated during and/or analyzed during this study are available from the corresponding author on reasonable request.
