# Supplementary material for: Advanced Messaging Intervention for Medication Adherence and Clinical Outcomes Among Patients With Cancer: Randomized Controlled Trial
Source: JMIR Cancer. 2023 Aug 31;9:e44612. doi: 10.2196/44612 (PMC10502590; doi:10.2196/44612)
Supplement: Multimedia Appendix 3 [file cancer_v9i1e44612_app3.docx]

**Supplementary material 3** The content of video is in Chinese. The following is the English translation.

‘Hello, I am a pharmacist from Shanghai Tenth People's Hospital. The chemotherapy pemetrexed you are taking can cause myelosuppression of hematotoxicity. To prevent and reduce the adverse effect, folic acid (FA) and vitamin B12 should be taken together with pemetrexed. Vitamin B12 is administered as an injection and only as prescribed at each hospital admission. FA tablets should be supplemented with 400μg daily starting one week before the dose of pemetrexed and continuing the same for at least 21 days beyond end of pemetrexed. Gold Theragran and Centrum are two multi-vitamins that contain FA and commonly used by adults in China. It should be noted that the content of FA in Gold Theragran is 400μg, which can be taken one capsule once a day. While the content of FA in Centrum is 200μg, you require to take two capsules once a day.’
